# Molecular mechanism of oroxyli semen against triple-negative breast cancer verified by bioinformatics and in vitro experiments

**DOI:** 10.1097/MD.0000000000034835

**Published:** 2023-09-15

**Authors:** Lulu Chen, Aishen Yang, Yangan Li, Xin Liu, Wei Jiang, Kehui Hu

**Affiliations:** a Clinical Laboratory of Zigong First People’s Hospital, Sichuan, China; b Department of Rehabilitation, Chishui People’s Hospital, Zunyi, China; c Department of Rehabilitation, Suining Central Hospital, Suining, China; d Daping Hospital, Army Medical University, Chongqing, China; e Department of Rehabilitation, Southwest Medical University, Sichuan, China.

**Keywords:** bioinformatics, chryseriol, oroxyli semen, TNBC

## Abstract

**Objective:**

This study aimed to use network pharmacology to predict the therapeutic mechanism of oroxyli semen (OS) on triple-negative breast cancer (TNBC) and validate it through in vitro experiments.

**Methods:**

The active ingredients and target proteins of OS were retrieved from the Traditional Chinese Medicine Systems Pharmacology database, and the TNBC-related target genes were obtained from the GeneCards database. The overlapping genes were used to construct a protein–protein interaction (PPI) network via the String database. Furthermore, we employed an online bioinformatics analysis platform (https://www.bioinformatics.com.cn/) to perform gene ontology and Kyoto Encyclopedia of Genes and Genomes pathway enrichment analyses to evaluate biological processes, molecular functions, and cellular components and generate simulated signal pathways. Additionally, molecular docking was used to evaluate the binding ability of small molecule drugs and signaling pathway targets. CCK8 assay was conducted to detect the effect of small molecule drugs on TNBC cell viability, and Western Blot was utilized to verify the expression of AKT, VEGF, and hypoxia-inducible factor 1-alpha (HIF-1α) proteins.

**Results:**

Fifteen active ingredients and 166 therapeutic targets of OS were obtained from the Traditional Chinese Medicine Systems Pharmacology database. The Venn diagram revealed that 163 targets were related to TNBC. The protein–protein interaction network analysis identified AKT1, IL-6, JUN, vascular endothelial growth factor A (VEGFA), CASP3, and HIF-1α as potential core targets through which OS may treat TNBC. Furthermore, the molecular docking results indicated that the active ingredient chryseriol in OS had good binding ability with VEGFA, and HIF-1α. CCK8 assay results indicated that chryseriol inhibited the viability of MDA-MB-231 and BT-20 cells. Western Blot demonstrated that chryseriol intervention led to a decrease in VEGFA, and HIF-1α protein expression compared with the control group (*P* < .05), increased the cleaved PARP.

**Conclusion:**

OS may exert its therapeutic effects on TNBC through multiple cellular signaling pathways. Chryseriol, the active component of OS, can enhance the apoptosis of TNBC cells by targeting VEGFA/HIF-1α pathway. This study provided new insights into the potential therapeutic mechanism of OS for TNBC and may aid in the development of novel therapeutic approaches for TNBC.

## 1. Introduction

Triple-negative breast cancer (TNBC) is a subtype of breast cancer that lacks expression of estrogen receptors, progesterone receptors, and human epidermal growth factor receptor 2.^[1]^ Its high metastatic potential and chemoresistance make it a challenging type of breast cancer to treat.^[2]^ Currently, research on TNBC mainly focuses on the molecular biology mechanisms of the disease and targeted therapies.^[3]^ Many studies use in vitro and in vivo models to evaluate the therapeutic effects of existing drugs and new compounds on TNBC.^[4]^ In addition, an increasing number of studies are focused on screening potential therapeutic drugs from traditional Chinese medicine and natural products, and predicting and validating their mechanisms of action using bioinformatics methods.^[5]^ Future research needs to continue to explore the molecular mechanisms of TNBC and find more effective treatment methods.

Oroxyli semen (OS) is a common traditional Chinese medicinal herb with a history spanning thousands of years. It contains various bioactive compounds, such as apigenin, luteolin, and quercetin, and exhibits diverse pharmacological effects including anti-inflammatory, antibacterial, lipid-lowering, and anticancer activities.^[6]^

Current research on OS primarily focuses on the extraction, isolation, and purification of its bioactive compounds, as well as investigating its pharmacological and anticancer effects.^[7]^ Among its bioactive compounds, apigenin is one of the most active and has been shown to inhibit the proliferation and migration of multiple tumor cells and induce tumor cell apoptosis.^[8]^ Although considerable progress has been made in OS research, due to its complex composition and diverse pharmacological mechanisms, further research is still needed to gain a deeper understanding of its impact on human health and to develop more effective treatment methods and applications.

## 2. Materials and methods

### 2.1. Data collection

The active constituents of drugs and corresponding therapeutic targets were retrieved from the Traditional Chinese Medicine Systems Pharmacology (TCMSP) database. The targets for TNBC were sourced from the GeneCards database. The cellular pathways were enriched by employing the gene ontology (GO) and Kyoto Encyclopedia of Genes and Genomes (KEGG) databases. The 2D structure of the small molecule drug chryseriol, which was utilized for molecular docking analysis, was obtained from the TCMSP database. The protein structures for vascular endothelial growth factor A (VEGFA) and hypoxia-inducible factor 1-alpha (HIF-1α) were sourced from the protein data bank database.

The TCMSP database (https://tcmspw.com/tcmsp.php) was used to retrieve information on the drug components and targets of OS. The aim was to screen for components with good oral bioavailability (OB ≥ 30%) and drug-likeness (DL ≥ 0.18) and predict their components and targets.

### 2.2. Obtaining targets information for TNBC

A search was performed on the GeneCards database (https://www.genecards.org/) using the keyword “TNBC” to obtain relevant targets information. Targets with a relevance score of at least 50 were selected to obtain more relevant disease targets.

### 2.3. Selection of common targets for drugs and diseases

Venn diagram generated using Venny 2.1 software was used to display the intersection of drug targets and disease targets. We selected the drug components and corresponding targets of OS from the TCMSP database, and obtained targets related to breast cancer treatment from the GeneCards database. We performed cross-analysis of these targets and selected the common targets for OS drugs and TNBC. Additionally, we constructed a protein–protein interaction network diagram of drugs and diseases using the STRING database to further analyze the interactions between these common targets.

### 2.4. Construction of PPI network and screening of core genes

We constructed a protein–protein interaction (PPI) network for the shared target genes of OS and breast cancer using the STRING database (https://str-db.org/cgi/input.pl). We selected the default settings and set the species as “Homo sapiens.” The results obtained from the STRING database were imported into Cytoscape 3.6.0, and then NetworkAnalyzer was used for topological analysis based on degree, betweenness centrality, average shortest path length, and closeness centrality. We sorted the genes by their degree and selected the top 20 genes with scores higher than the average score as key targets. Ultimately, we identified 163 key targets and plotted them as a graph based on their respective values.

### 2.5. GO database enrichment analysis

We conducted GO enrichment analysis on the shared targets of OS and triple-negative breast cancer, using the GO database to analyze biological processes, molecular functions, and cellular components. We filtered the enriched pathways based on a corrected *P*-value ≤ 0.05. An online analysis platform (https://www.bioinformatics.com.cn/) was used to list the top 5 gene enrichments for biological processes, molecular functions, and cellular components.

### 2.6. KEGG database enrichment analysis

We conducted KEGG pathway enrichment analysis on the common targets of OS and triple-negative breast cancer using the David database. We filtered out enriched pathways based on a corrected *P*-value of ≤.05. Subsequently, we annotated the pathways in which each gene was involved using a bioinformatics online analysis platform to further investigate the association between these pathways and the diseases.

### 2.7. Construct OS-component-TNBC target network

To better elucidate the complex interaction between OS components and breast cancer and their corresponding target genes, we constructed an OS component-TNBC target network based on the corresponding components and disease target genes included in this study. The network was visualized using Cytoscape 3.6.0. The components were sorted based on their degree values, with higher values indicating greater importance.

### 2.8. Molecular docking

We used AutoDock software for molecular docking. Firstly, we obtained the 3D structures of the required protein and ligand molecules from the protein data bank database (https://www.rcsb.org/). To meet the requirements of AutoDock, we converted the pdb files into pdbqt format files and added hydrogen atoms and calculated charges. Next, we configured the AutoDock parameters according to our needs, including defining the grid box, selecting the search algorithm, setting the search space, and energy function. Defining the grid box is an important step that determines the size of the search space and the position of the ligand molecule. Selecting the appropriate search algorithm and energy function also have a significant impact on the accuracy of the results. After the configuration is complete, AutoDock scans the ligand molecule, searches all possible positions, and calculates the interaction energy at each position. After docking, we can analyze and visualize the results, such as calculating the interaction energy between the ligand and protein, and drawing energy contour maps. These results can provide important references for subsequent drug design and optimization.

### 2.9. Cell culture

MDA-MB-231 cells were cultured in DMEM medium (with high glucose) and 10% fetal bovine serum (FBS). BT-20 cells were cultured in RPMI 1640 medium and 10% FBS. 1% penicillin–streptomycin (PPS) was added to avoid bacterial infection during cell culture. Cells were cultured at 37 °C and 5% CO_2_ and the next step was performed when the cells reached approximately 70% to 80% confluency.

### 2.10. CCK8

MDA-MB-231 and BT-20 breast cancer cells (5 × 10^3^ cells per well) were seeded into a 96-well plate. Different concentrations of OS (0, 5, 15, and 20 μmol/L) were added to the treatment and control groups, followed by incubation for 48 hours at 37 °C and 5% CO_2_. Cell viability was measured using the Cell Counting Kit-8 (CCK-8, Bimake, Houston, TX). CCK-8 reagent was added to each well and incubated for 1 to 4 hours in a cell culture incubator. Absorbance at 450 nm was measured using a microplate reader (BioTek Instruments, Winooski, VT) to assess cell viability.

### 2.11. Western blot

To prepare for protein extraction, cells were divided into 2 groups: the control group with no added OS (0 μmol/L) and the experimental group with 20 μmol/L OS added. After 48 hours of incubation, the culture medium was removed and the cells were washed 3 times with sterile PBS buffer. Then, pre-cooled protein lysis buffer containing 10% phosphatase inhibitor was added to the cells, and the cells were scraped with a cell scraper and collected into a 1.5 mL EP tube. The tube was placed on ice and the cells were lysed for 20 minutes. After lysis, an appropriate amount of loading buffer was added, and the sample was heated at 95 °C for 10 minutes and stored for later use. The proteins were separated by 10% polyacrylamide gel electrophoresis and transferred onto a PVDF membrane (Millipore, Billerica, MA) when the bromophenol blue was close to the bottom of the gel. The membrane was then blocked with 10% skim milk at room temperature for 30 minutes, followed by overnight incubation with the primary antibody at 4 °C. The next day, the membrane was washed 3 times with TBST buffer and incubated with a goat anti-rabbit or anti-mouse secondary antibody labeled with horseradish peroxidase at 37 °C for 1 hour.

### 2.12. Statistical analysis

The data from all samples were presented as mean ± standard deviation (SD) after being measured 3 times. Single-factor analysis of variance (ANOVA) was conducted using Graphpad Prism 9.0 software to compare the differences among different groups. A difference was considered statistically significant when the *P*-value was <0.05.

## 3. Results

### 3.1. Obtaining common targets for OS therapy in TNBC

**Figure 1.**
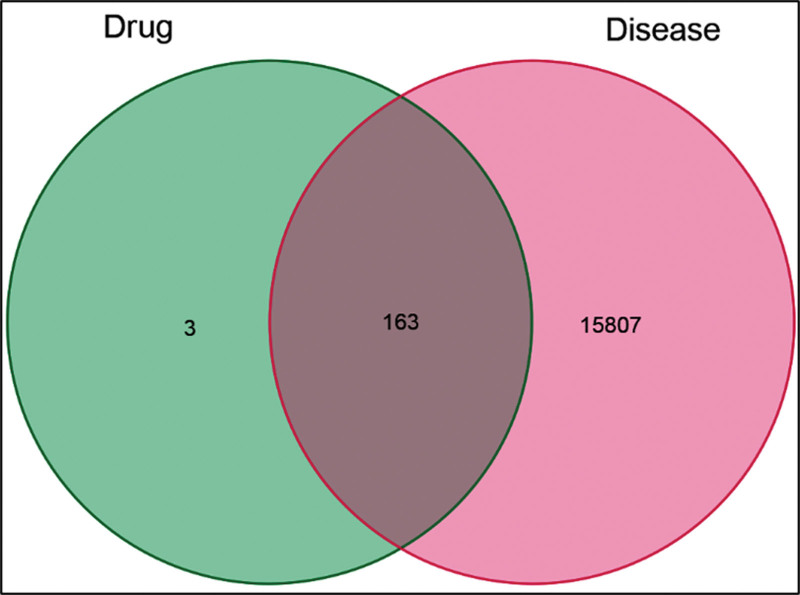
Venn diagram of OS and TNBC. The red color represents the targets of TNBC, the green color represents the targets of OS, and the dark red color in the middle represents the common targets.

**Figure 2.**
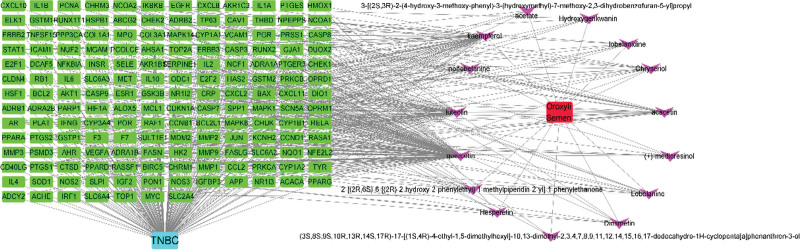
Network of active ingredients and targets of OS. The green nodes represent the target genes of TNBC, and the purple nodes represent the active ingredients.

In this study, we applied specific screening criteria for drug components in the TCMSP database, selecting components with drug-likeness (DL) and oral bioavailability (OB) and only retaining those with OB >30% for further analysis. Following these criteria, we identified 15 effective components and 166 targets. We then associated these targets with breast cancer-related genes, resulting in 15,960 genes associated with TNBC. By intersecting the drug target genes with TNBC-related target genes, we ultimately obtained 163 common targets. We used Venny 2.1 software to draw a Venn diagram (Fig. 1) and Cytoscape 3.7.0 software to draw a network diagram of OS components-TNBC targets (Fig. 2).

### 3.2. Constructing PPI network and screening of core genes

**Figure 3.**
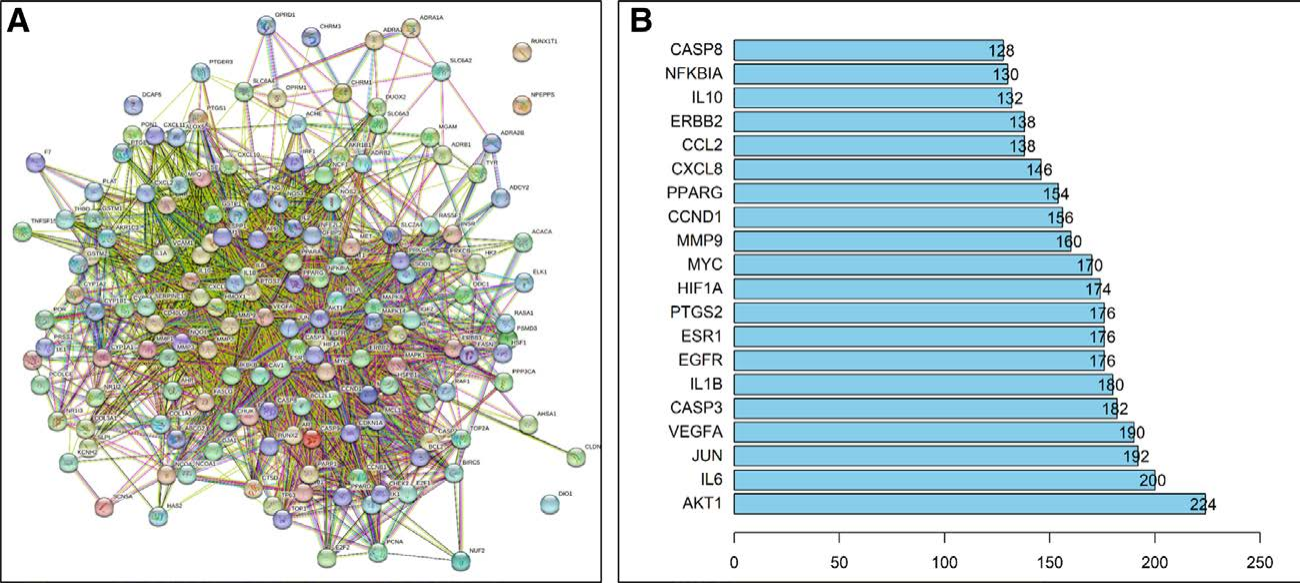
Constructing PPI network and selecting core genes: (A) PPI network diagram of OS-TNBC common targets. PPI = protein-protein interaction. (B) Twenty key targets selected by PPI topology analysis based on degree. The horizontal axis represents the value of degree, and the vertical axis represents the specific target name.

In our study, we imported the common target genes of OS and TNBC into the STRING database, setting the species as human and using default settings for other parameters. As a result, we obtained a PPI network diagram (Fig. 3A). Subsequently, we performed topological analysis on the PPI network using Cytoscape 3.6.0 software and selected the top-scoring genes with a score >75 based on their degree centrality as key targets. Finally, we identified the top 20 key targets (Fig. 3B), with AKT1, IL-6, and JUN being the top 3 targets in descending order of degree centrality.

### 3.3. Biological function and pathway enrichment analysis

#### 3.3.1. GO enrichment analysis.

**Figure 4.**
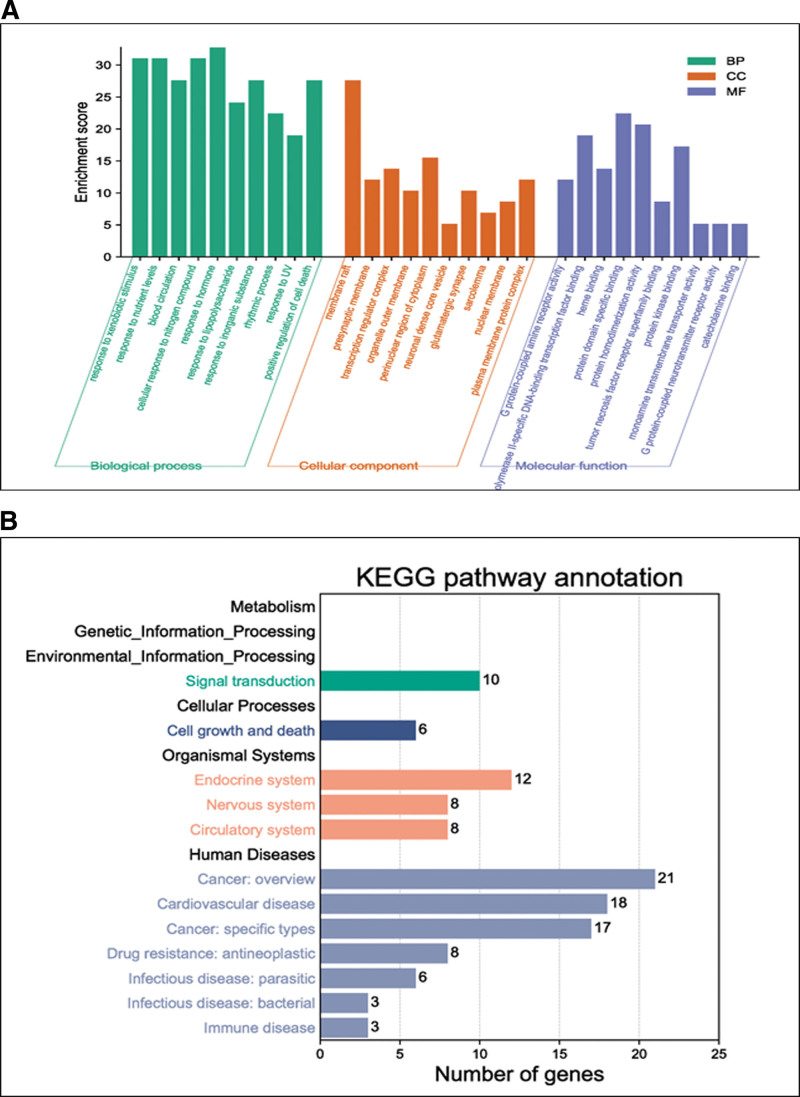
Functional enrichment analysis: (A) GO enrichment analysis. (B) KEGG enrichment analysis.

By performing an enrichment analysis of the intersection genes between OS and triple-negative breast cancer (TNBC) using the GO database, we found that the core biological processes of OS treatment for TNBC are mainly related to xenobiotic stimulus, nutrient levels, cellular response to nitrogen compounds, response to hormones, and response to inorganic substances. These processes are primarily involved in cellular components, such as the membrane raft, perinuclear region of the cytoplasm, transcription regulator complex, and plasma membrane protein complex. The core biological processes are completed through molecular functions, including protein domain-specific binding, protein homodimerization activity, polymerase II-specific DNA-binding transcription factor binding, and protein kinase binding (Fig. 4A).

#### 3.3.2. KEGG enrichment analysis.

**Figure 5.**
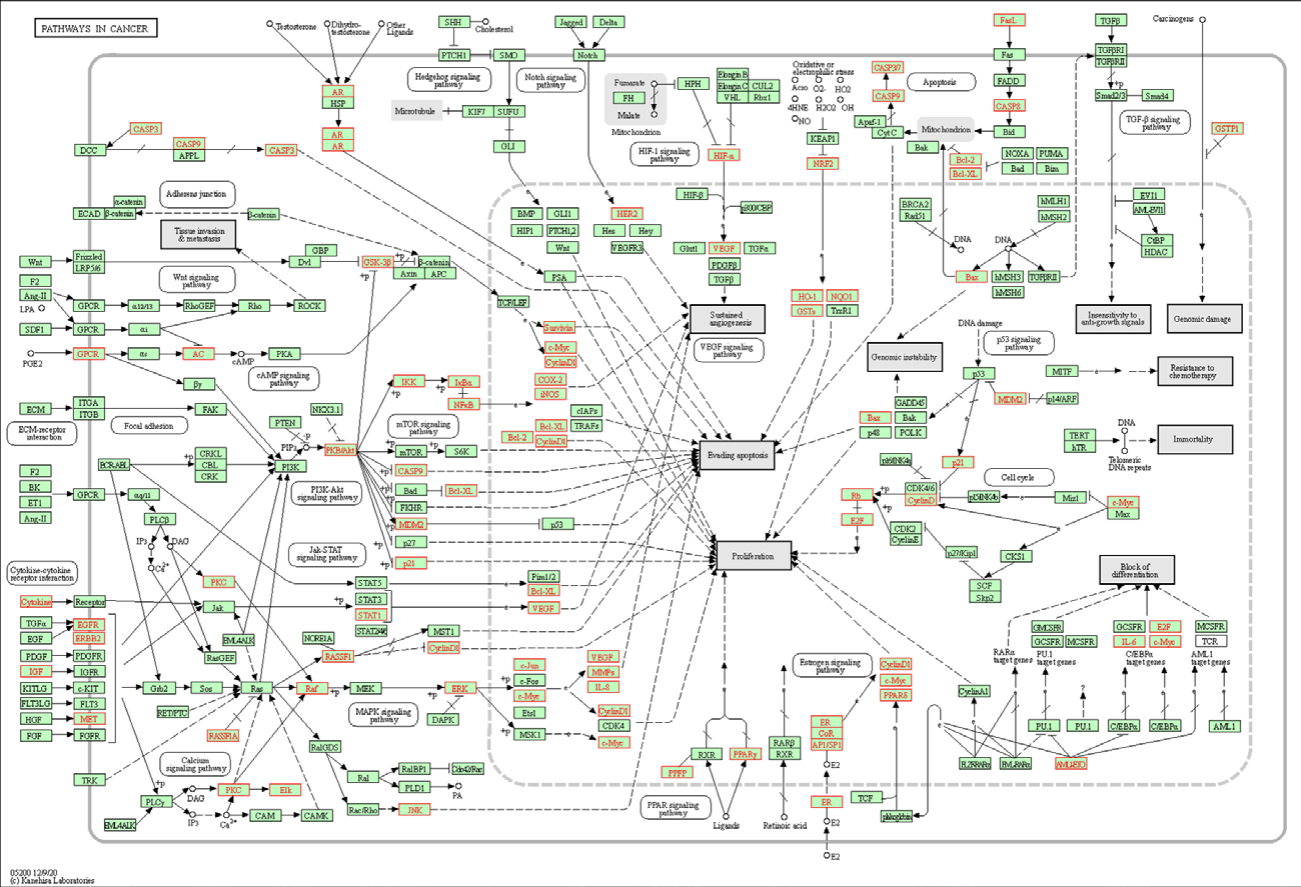
KEGG simulated signaling pathway analysis.

The KEGG enrichment analysis revealed that the intersection genes between OS and TNBC are primarily enriched in processes related to cancer, signal transduction, endocrine system, and cardiovascular disease (Fig. 4B). Based on the KEGG analysis of the simulated signaling pathway, it was discovered that the classical PI3K/AKT signaling pathway continues to play a crucial role and significantly impacts cell proliferation and growth. In the signaling pathway diagram, we found that OS may induce cancer cell apoptosis by inhibiting the VEGFA/HIF-1α pathway (Fig. 5). Therefore, we further performed molecular docking analysis and in vitro experimental verification.

### 3.4. Molecular docking

**Figure 6.**
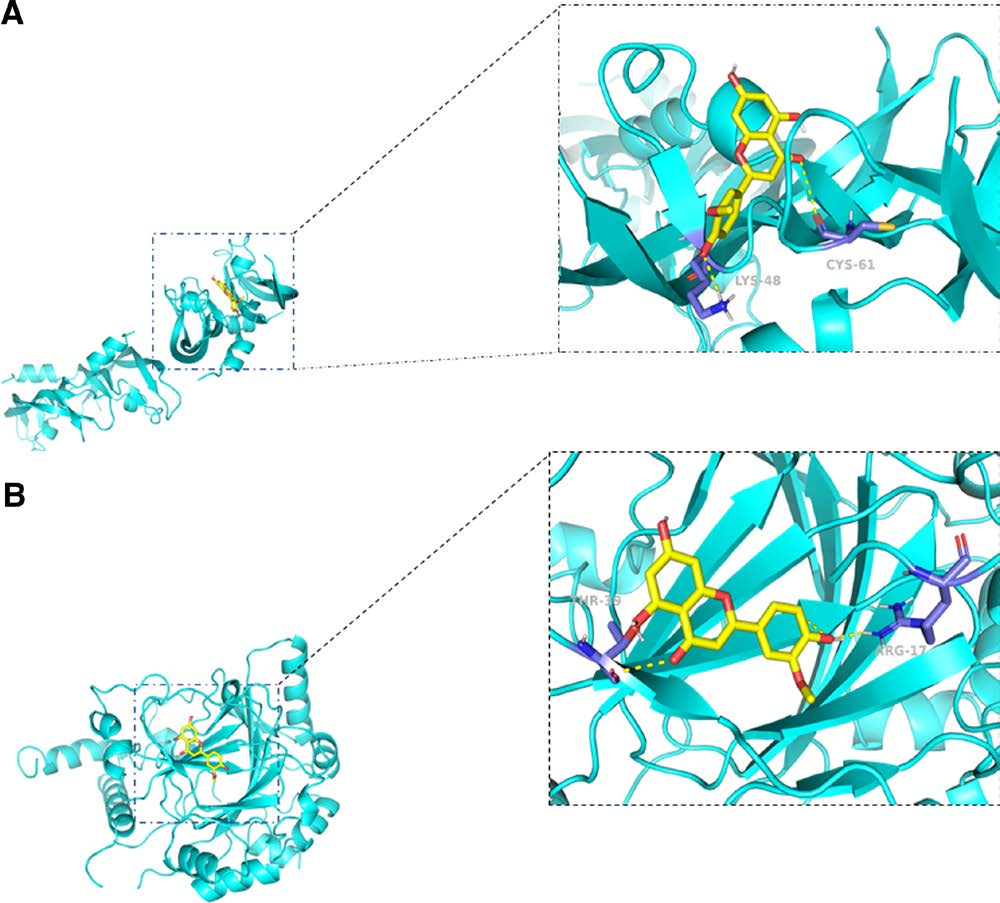
Molecular docking. (A) Molecular docking model of chryseriol with VEGFA. (B) Molecular docking model of chryseriol with HIF-1α.

To investigate whether the biological activity of chryseriol is related to the VEGFA and HIF-1α signaling pathways, we used molecular docking to evaluate its binding to the key targets in these pathways. The research results showed that hydrogen bonding is the main mechanism for the interaction between the targets and chryseriol, and a binding energy <0 indicates better docking efficiency. Specifically, we found that chryseriol can bind to VEGFA and HIF-1α, with binding energies of −7.0 kcal/mol and −7.4 kcal/mol, respectively. This research result suggests that chryseriol may exert its anticancer effects through the VEGFA/HIF-1α signaling pathway (Fig. 6).

### 3.5. Chryseriol can induce apoptosis of human TNBC cells by inhibiting VEGFA/HIF-1α pathway

**Figure 7.**
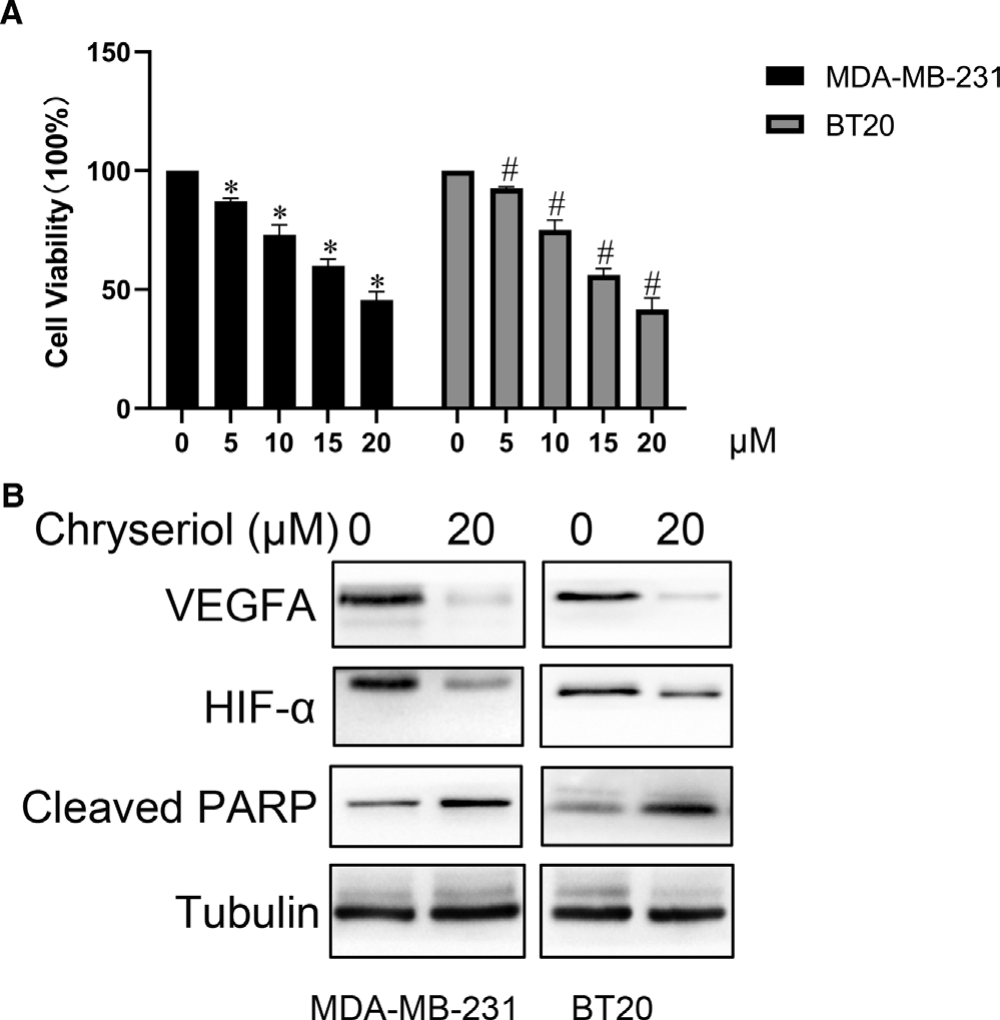
In vitro experiments. (A) Cell viability assay. MDA-MB-231, BT20 cells were treated with 0, 5, 10, 15, 20 μmol/L chryseriol for 48 hours, and cell survival rate was detected by CCK-8 assay. ^*^P < .05, compared with 0 μmol/L group, ^#^*P* < .05, compared with 0 μmol/L group. (B) Western blot showed that the expression of VEGFA, HIF-1α and cleaved PARP were decreased after treatment of MDA-MB-231 and BT20 cells with 20 μmol/L chryseriol for 48 hours.

CCK8 assay results showed that chryseriol could inhibit the viability of MDA-MB-231 and BT20 cells, and this inhibitory effect was dose-dependent within 48 hours. As the drug concentration increased, the cell survival rate continued to decrease. The high concentration of chryseriol (20 μmol/L) significantly reduced the survival rate of the 2 triple-negative breast cancer cells when compared to the control group treated with 0 μmol/L chryseriol (*P* < .05) (Fig. 7A).

To further confirm the anti-TNBC mechanism of chryseriol obtained from KEGG enrichment analysis and molecular docking, western blot analysis was conducted to detect the expression of pathway proteins in 2 TNBC cell lines treated with 0 μmol/L and 20 μmol/L chryseriol for 48 hours. The results showed that treatment with chryseriol (20 μmol/L) for 48 hours led to a decrease in the protein levels of VEGFA and HIF-1α (*P* < .05) and increased the expression of the cleaved PARP (Fig. 7B).

## 4. Discussion

OS, as a traditional Chinese medicine, has the effects of moistening the lungs, benefiting the throat, soothing the liver, and harmonizing the stomach. It is mainly used for lung heat cough, aphonia, throat swelling and pain, liver and stomach qi pain, and sores and ulcers. Modern research has found that OS has various pharmacological effects, such as antibacterial, anti-inflammatory, anti-allergic, antiviral, antioxidant, antitumor, and hypoglycemic effects. Through the TCMSP database, we have obtained 15 active ingredients and 166 therapeutic targets of OS. We found that 163 of the drugs were matched by the TNBC treatment target, by taking the intersection of the TNBC related therapeutic targets obtained from Genecards.

Through the network diagram of OS components-TNBC targets, we found that the main anti-TNBC components of OS are quercetin, luteolin, kaempferol, chryseriol, and acacetin. A large number of literature reports have already investigated the antitumor properties of components such as quercetin, luteolin, and kaempferol. As chryseriol possesses strong antioxidant and anti-inflammatory capabilities, in this study, we aim to explore the antitumor properties of chryseriol. Through the gene enrichment analysis using the GO database, we found that OS mainly exerts its effects through biological processes such as xenobiotic stimulus, nutrient levels, cellular response to nitrogen compounds, response to hormones, and response to inorganic substances. The cellular components involved primarily include membrane raft, perinuclear region of the cytoplasm, transcription regulator complex, and plasma membrane protein complex. Furthermore, it achieves its anti-TNBC action through mechanisms such as protein domain-specific binding, protein homodimerization activity, polymerase II-specific DNA-binding transcription factor binding, and protein kinase binding. Literature reported chrysoeriol can bind to the protein kinase domain of Src and inhibit p-Src in melanoma cells and tissues, thus participating in the growth and metastasis of melanoma.^[9]^ Simultaneously, chrysoeriol can slow down the progression of OVA-induced asthma in mice by inhibiting NF-κB/HIF-1α and MAPK/STAT1 pathways, which is consistent with the GO enrichment results.^[10]^

In the present research, the KEGG database was employed for pathway enrichment analysis. It was observed that within the simulated pathway, VEGFA and HIF-1α serve as crucial nodes in combating TNBC cell pathways. A multitude of studies suggest that VEGFA also functions as an immunosuppressive factor, playing a vital role in the process of tumor angiogenesis.^[11]^ In hosts bearing tumors, VEGFA is capable of modulating immune cells (DC, MDSC, TAM), inducing the accumulation of regulatory T cells while concurrently suppressing T cell functionality.^[12]^ Utilizing antiangiogenic agents that target VEGFA/VEGFR may assist in restricting immunosuppression induced by tumors.^[13]^ HIF-α, a subunit of the HIF-1 transcription factor, is encoded by the HIF1α gene.^[14]^ It contributes to cancer development by facilitating the shift from oxygen-reliant ATP generation to glycolysis,^[15]^ and is linked to genetic instability,^[16]^ inflammation^[17]^ that promotes tumor growth,^[18]^ and evasion of the immune system.^[19]^ The molecular docking results demonstrate that chryseriol has a good binding ability with proteins VEGFA and HIF-α. The CCK8 assay confirmed that chryseriol indeed exerts an inhibitory effect on the viability of TNBC cells. Meanwhile, Western Blot experiments demonstrated that in MDA-MB-231 and BT-20 cells, the expression of VEGFA and HIF-1α proteins was significantly suppressed at a chryseriol concentration of 20 μmol/L, while the expression of Cleaved PRAP increased. The results suggest that chryseriol may induce TNBC cell apoptosis by inhibiting the VEGFA/HIF-1α pathway. However, additional experiments are necessary to determine its potential cytotoxicity and to validate its efficacy in vivo.

## 5. Conclusion

OS has a strong therapeutic effect in combating TNBC, with most of its therapeutic targets directed towards TNBC. Its main active ingredient, chryseriol, may promote TNBC cell apoptosis by inhibiting the VEGFA/HIF-1α pathway. This article proposes a relatively new view on the therapeutic mechanism of the Chinese herbal medicine OS, providing further evidence for its clinical use.

## Author contributions

**Conceptualization:** Wei Jiang, Kehui Hu.

**Data curation:** Lulu Chen, Aishen Yang.

**Investigation:** Yangan Li, Xin Liu.

**Software:** Yangan Li, Xin Liu.

**Visualization:** Yangan Li, Xin Liu.

**Writing – original draft:** Lulu Chen, Aishen Yang.

**Writing – review & editing:** Wei Jiang, Kehui Hu.
